# Novel pleiotropic effects of bioactive phospholipids in human lung cancer metastasis

**DOI:** 10.18632/oncotarget.17461

**Published:** 2017-04-27

**Authors:** Gabriela Schneider, Zachariah Payne Sellers, Kamila Bujko, Sham S. Kakar, Magda Kucia, Mariusz Z. Ratajczak

**Affiliations:** ^1^ Stem Cell Institute at James Graham Brown Cancer Center, University of Louisville, Louisville, Kentucky, USA; ^2^ Department of Physiology and James Graham Brown Cancer Center, University of Louisville, Louisville, Kentucky, USA; ^3^ Department of Regenerative Medicine, Medical University of Warsaw, Warsaw, Poland

**Keywords:** bioactive phospholipids, priming, HGF/SF, lung cancer, metastasis

## Abstract

We previously proposed that one of the unwanted side effects of chemotherapy and radiotherapy is the increase in several peptide- and non-peptide based chemoattractants in damaged tissues, leading to induction of a prometastatic microenvironment for remaining cancer cells. Herein, we turned out our attention to a potential role of bioactive phospholipids (BphsLs), such as sphingosine-1-phosphate (S1P), ceramide-1-phosphate (C1P), lysophosphatidylcholine (LPC), and lysophosphatidic acid (LPA) in lung cancer (LC) metastasis. We report that LC cells express several functional BphL receptors (for S1P, LPC, and LPA) as well as several enzymes involved in their metabolism and that BphsLs are potent chemokinetic and adhesion factors for these cells. We also demonstrate for the first time the novel role of C1P as a prometastatic factor in LC cells. In addition to their chemokinetic activities, BphsLs also sensitize or prime the chemotactic responsiveness of LC cells to known prometastatic factors such as hepatocyte growth factor/scatter factor (HGF/SF). Thus, for the first time we demonstrate a prometastatic effect that is based on the priming of a cell's responsiveness to chemotactic factors by chemokinetic factors. To our surprise, none of the bioactive lipids induced proliferation of LC cells or ameliorated toxic effects of vincristine treatment. Interestingly, BphsLs increase adhesion of LC cells to bone marrow-derived stromal cells and stimulate these cells to release ExNs, which additionally increase LC cell motility. In conclusion, our results show that BphsLs are important modulators of prometastatic environment. Therefore, their inhibitors could be considered as potential anti-metastatic drug candidates to be included as a part of post radio- and/or chemo- therapy treatment.

## INTRODUCTION

Metastasis is responsible for more than 90% of cancer-associated mortality, and therefore the need to prevent metastasis is one of the therapeutic priorities in clinical oncology [1]. It is well known that lung cancer (LC) is highly metastatic, and when diagnosed with distant metastases, the predicted 5-year survival rate is only 4% [2]. Several chemoattractants that could direct metastasis of LC include hepatocyte growth factor/scatter factor (HGF/SF) [3], the α-chemokine stromal-derived factor 1 (SDF-1) [4], monocyte chemoattractant protein 1 (MCP1) [5], and chemokine (C-C motif) ligand 19 (CCL19) [6]. Interestingly, what is not often emphasized, these chemoattractants for LC cells are detected in biological fluids and tissues at much lower concentrations than those employed routinely in assays to assess the migration of LC cells [7–9]. This was the reason why we began a search for other more relevant chemoattractants that could induce metastasis of these cells in *in vivo* situations.

Recently, we identified extracellular nucleotides (ExNs) as potent stimulators of LC cell migration [10]. However, taking into account the fact that ExNs are rapidly degraded by ExN-processing enzymes [11, 12], we turned our attention to bioactive phospholipids (BphsLs), such as sphingosine-1-phosphate (S1P), ceramide-1-phosphate (C1P), lysophosphatidylcholine (LPC), and its derivative lysophosphatidic acid (LPA), as candidate stimulators. It is known that BphsLs activate several G-protein coupled receptors expressed on tumor cells. While S1P activates S1PR1-R5 receptors, LPA interacts with LPAR1–5 type receptors and LPC activates G2A and GPR4 receptors. Somehow, surprisingly the binding receptor/s for a very potent bioactive phospholipid chemotractant - that is C1P, have not been identified yet.

In our previous work we demonstrated that all these BphsLs increase metastatic potential of human rhabdomyosarcoma cells [13, 14]. Nevertheless, it is important to keep in mind that the role of BphsLs in cancer metastasis is pleiotropic. These bioactive molecules not only interact with their specific receptors on cancer cells but also affect biology of endothelial cells, tumor associated fibroblasts and may modulate anti-tumor response of immune cells [15].

It is well known that S1P is secreted from several types of cells, which explains its relatively high (micromolar) concentration in peripheral blood and lymph [15]. Similarly, the concentration of C1P, LPA, and LPC are also comparably high in peripheral blood. In addition to steady-state conditions, all of these BphsLs, like ExNs, are also released from “leaky” damaged cells [10, 13–18].

Based on the latter findings, we recently proposed that one of the unwanted side effects of radio- and/or chemo- therapy is the induction of a prometastatic microenvironment in healthy normal collateral tissues as the result of damage from anti-cancer treatment [10, 13, 14]. Our studies indicate that increased levels of ExNs and BphsLs here play an important role [10, 13, 14].

Since BphsLs, in particular C1P, LPA and LPC, have not been well studied as direct chemoattractants for LC cells, we decided to fill these gaps in our knowledge. We therefore characterized their effects on LC cell migration, adhesion, and stromal-LC cell interactions. We found that BphsLs are involved in direct and indirect pleiotropic mechanisms involved in LC metastasis. Therefore, our results show BphsLs to be important modulators of a prometastatic environment, and their therapeutic inhibition should be considered as a supportive part of post radio- and/or chemo- therapy treatment. This however requires further studies.

## RESULTS

### Human LC cells express several functional receptors for BphsLs

We have already reported that radio- and chemo- therapy increases the levels of S1P, C1P, LPA, and LPC in murine organs and enhances the prometastatic potential of human rhabdomyosarcoma cells [13, 14]. Here we asked whether a similar mechanism also occurs in human LC cells, and we first evaluated mRNA expression for S1P, LPA, and LPC receptors. However, since the C1P receptor has not yet been cloned, we were not able to investigate its expression [19]. Moreover, since the pro-migratory effect of LPC is assigned to LPA, which is derived from LPC in an autotaxin (ATX)-dependent manner [20], and since, in addition to “classical” LPA receptors, LPC activates G2A and GPR_4_ receptors, we also evaluated the expression of the mRNAs for ATX, G2A, and GPR_4_ in human LC cells [21, 22]. In parallel, we also focused on the expression of mRNAs that encode enzymes involved in the synthesis and degradation of BphsLs [20, 23–27].

**Figure 1:**
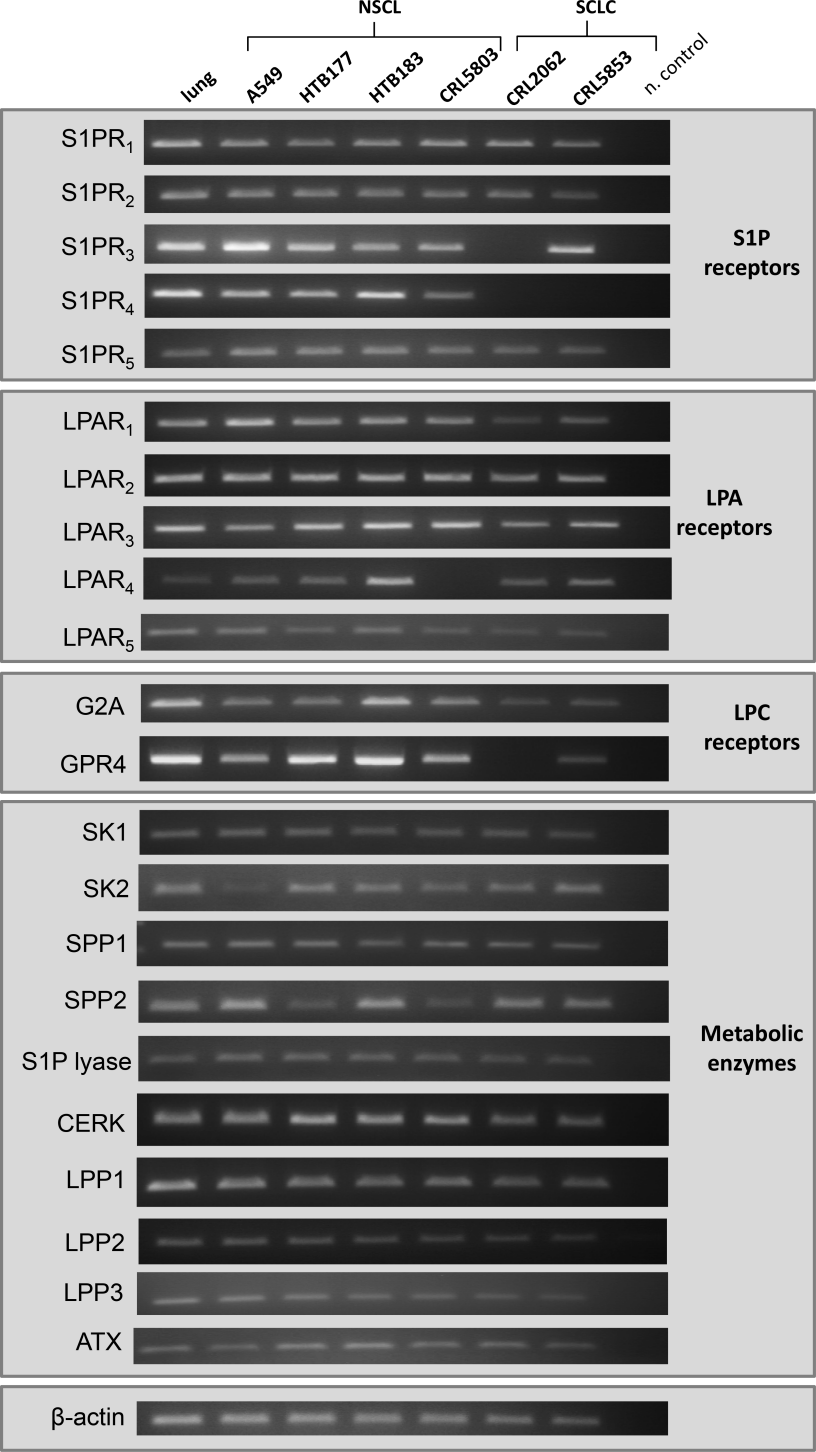
Expression of receptors and enzymes involved in BphsL signaling and synthesis RT-PCR analysis of the expression of S1P, LPA, and LPC receptors as well as enzymes involved in the synthesis and degradation of the tested BphsLs.

We performed studies on four NSCLC and two SCLC human cell lines as well as on normal lung tissue, and as it is shown in Figure 1, we observed that S1P receptors (S1PR1-R5), LPA receptors (LPAR1-R5) as well as GPR4 and G2A for LPC are highly expressed by most of the LC cell lines. We also found that the LC cell lines investigated herein express mRNAs for several enzymes involved in BphsLs metabolism, including ATX, an enzyme regulating synthesis of LPA from LPC [20]. Interestingly, in parallel studies we also detected in normal lung tissue expression of BphsLs receptors as well as enzymes involved BphsLs metabolism.

**Figure 2:**
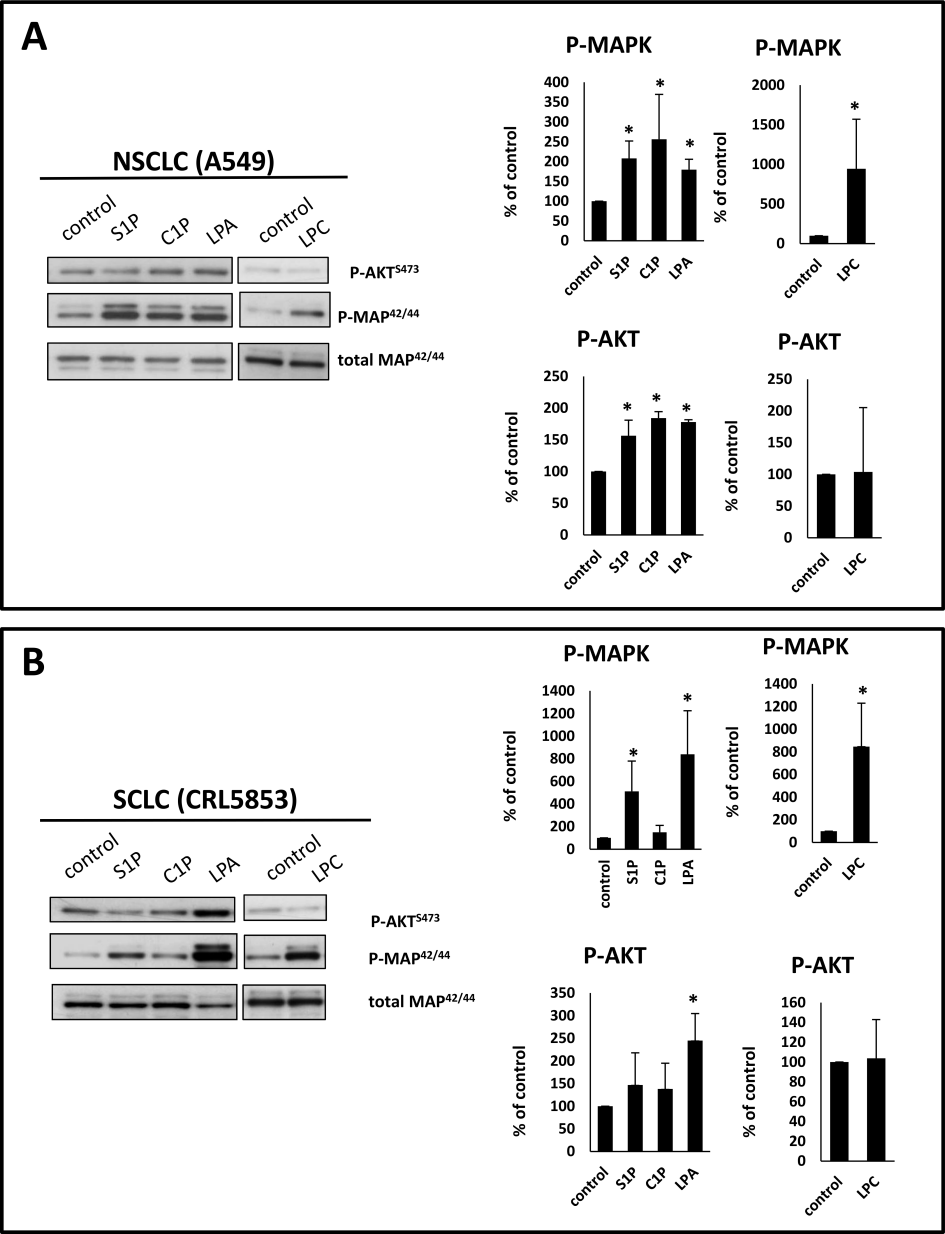
BsphL receptors in lung cancer cells are functional Phosphorylation of p42/44 MAPK and AKT in human NSCLC (Panel **A**) and SCLC (Panel **B**) cell lines stimulated for 5 min with S1P (1 μM), C1P (0.5 μM), or LPA (0.1 μM) or stimulated for 2 h with LPC (20 μM). Representative WB (left) and densytometric analysis (right graphs) from three independent analysis are presented *p ≤ 0.05.

Moreover, by employing AKT and p42/44 MAPK phosphorylation studies we found that S1P, C1P, LPA, and LPC activate phosphorylation of p42/44 MAPK in the NSCLC cell line A549 and in the SCLC cell line CRL5853 (Figure 2). LPA also stimulated phosphorylation of AKT in both cell lines (Figure 2). Interestingly, phosphorylation of p42/44 MAPK by LPC required much longer incubation times, which suggests that it was in fact the result of conversion of LPC to LPA and not a direct stimulation of classical LPC receptors, which are expressed at a low level in these cell lines (Figure 1).

### The bioactive phospholipids tested in our study affect neither proliferation nor survival of LC cells but stimulate cell migration and adhesion

BphsLs have been reported to stimulate proliferation in a range of normal and malignant cells [28–33]. To our surprise, however, we did not observe any effect on the proliferation of LC cell lines used in our studies when BphsLs were added to the medium in physiologically relevant concentrations (Supplementary Figure 1). Of note, because of cell toxicity, LPC was employed at a lower concentration. Moreover, BphsLs did not enhance in our hands the survival of LC cells cultured under serum-free conditions or in the presence of vincristine (Supplementary Figure 2).

**Figure 3:**
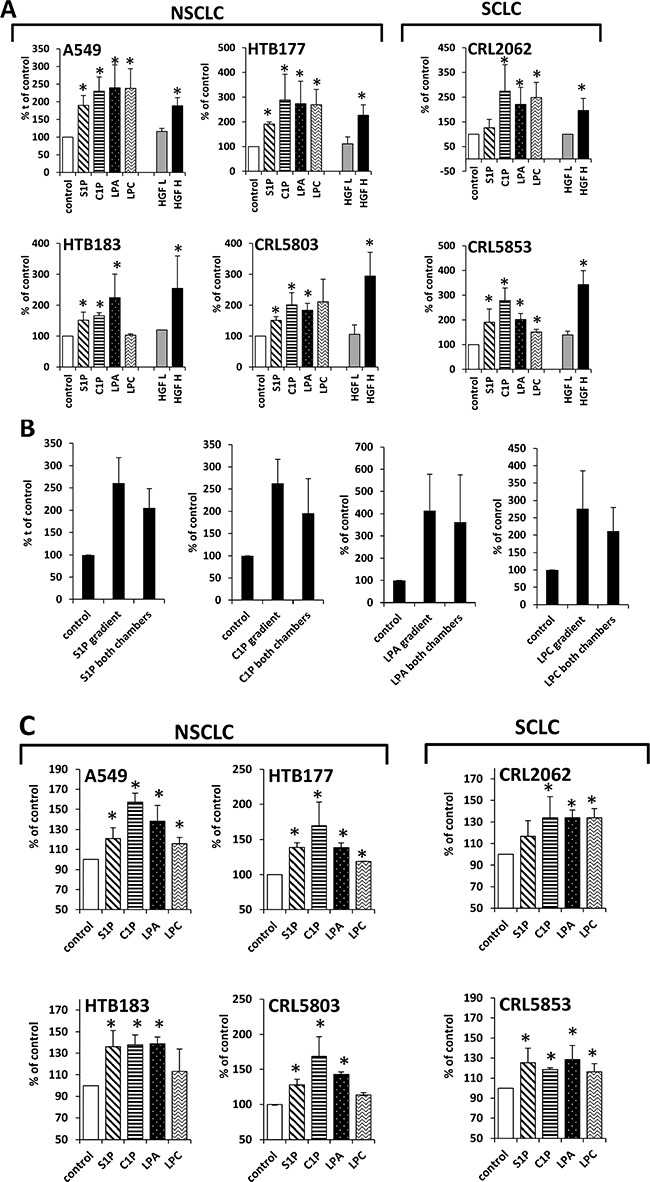
BphsLs induce migration of LC cells (Panel **A**) Responsiveness of NSCLC and SCLC cells to gradients of S1P (1 μM), C1P (0.5 μM), LPA (0.1 μM), or LPC (20 μM) compared with the known chemoattractant HGF employed at supraphysiological (H; 10 ng/ml) and physiological (L; 0.3 ng/ml) concentrations. The experiment was performed at least twice in duplicate, *p ≤ 0.05. (Panel **B**) Chemotaxis versus chemokinesis analysis by comparing the migration of A549 cells in response to BphsL gradients (BphsL in the lower chamber) or no gradient (BphsL in both chambers). The experiment was performed twice in duplicate, *p ≤ 0.05. (Panel **C**) Adhesion of LC cells to fibronectin. The cells were unstimulated (control) or stimulated with S1P (1 μM), C1P (0.5 μM), LPA (0.1 μM), or LPC (20 μM) for 10 min. The number of adherent cells was measured by microscopic analysis. Data from two separate experiments are pooled together, and means ± SD are shown, *p ≤ 0.05.

Next we analyzed whether tested BphsLs can induce cell migration. To address this, we employed Transwells which consist of two chambers separated by the membrane with appropriate pore size that allows cells to migrate from upper to lower chamber. Cells were seeded on the upper chambers and studied BphsLs as chemoattractants were added to the lower chambers. Next cells were allowed to migrate for 24h after which non-migrated cells were removed from the top of the membrane and migrated cells that were localized on the bottom of the membrane were fixed, stained and counted under microscope. We found that all tested BphsLs strongly enhanced migration of NSCLC and SCLC cells (Figure 3A). Importantly, the response of cells to BphsLs was stronger than to physiological concentrations of hepatocyte growth factor/scatter factor (HGF/SF), which is a well-known chemoattractant for these cells. However, as shown in Figure 3, HGF/SF was more effective in these assays when employed at supra-physiological concentrations.

To check whether BphsLs induce direct (chemotaxis) or indirect (chemokinesis) movement of cells, we compared the migration of A549 cells in the Transwells by employing a checkerboard assay [13, 14], which was performed in the presence of BphsLs added into the lower chamber only (creating BphsLs gradient) or added simultaneously to the upper and lower chambers (no BphsLs gradient). Figure 3B indicates that all tested BphsLs induced mostly random movement of cells (chemokinesis) rather than gradient-directed chemotaxis. Figure 3C also demonstrates that all BphsLs promoted adhesion of LC cells to fibronectin-coated plates.

### BphsLs, as chemokinetic factors, sensitize or prime the chemotactic responsiveness of LC cells to an HGF/SF gradient

The migration of cells is stimulated by both chemokinetic and chemotactic factors [34]. However, while the former process plays a role in the migration of cells in all directions, the latter process directs their movement along a chemoattractant gradient [34]. We have hypothesized that, by increasing the overall motility of cells, chemokinetic factors sensitive or prime the migration of cells to move up the gradient of a chemoattractant.

**Figure 4:**
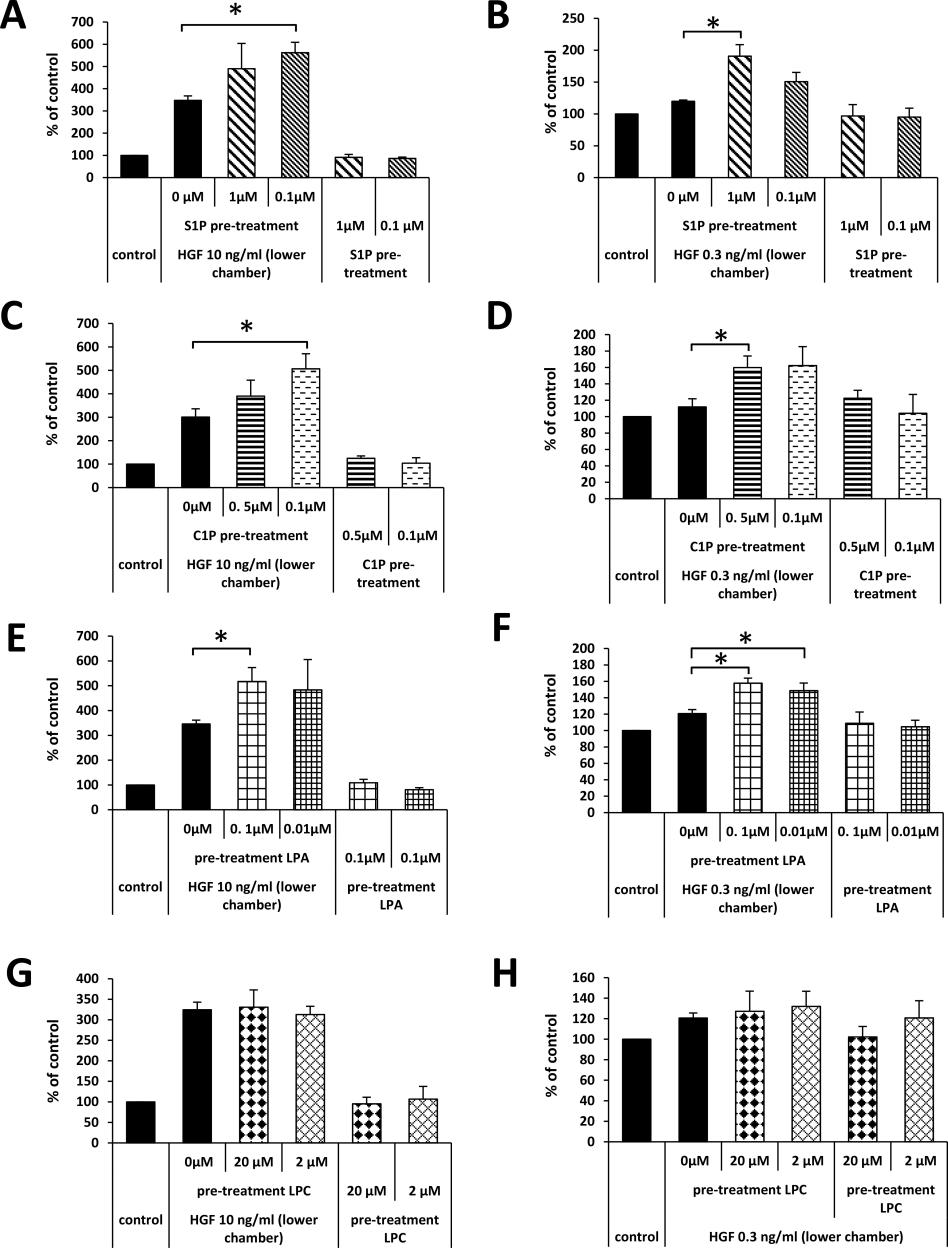
Pretreatment of A549 cells with BphsLs regulates their response to HGF The effect of pretreatment of A549 cells with S1P (Panels **A** and **B**), C1P (Panels **C** and **D**), LPA (Panels **E** and **F**), or LPC (Panels **G** and **H**) on the migration of cells in response to HGF employed at supraphysiological (Panel A, C, E, and G) or physiological (Panels B, D, F, and H) concentrations. The experiment was performed at least twice in duplicate, *p ≤ 0.05.

To test this novel hypothesis, we exposed A549 cells for 30 minutes to different doses of BphsLs and loaded the cells into the upper chamber of a Transwell system. The lower chambers were filled with medium with or without HGF, employed at supra-physiological (Figure 4A–4G) or physiological (Figure 4B–4H) concentrations.

As shown in Figure 4, pre-treatment of LC cells did not affect their migration in response to control medium (RMPI + 0.5% BSA). Moreover, as shown in Figure 4B–4H and similarly as in Figure 3A, we did not observe a significant increase in LC cell migration up a shallow physiological HGF gradient. By contrast, when BphsL-pretreated cells were tested in a migration assay in response to HGF/SF gradients, we observed an increase in chemotactic migration both to high (supra-physiological) and, more importantly, to low (physiological) concentrations of this chemoattractant in the lower chamber of the Transwell system. However, to our surprise, in contrast to S1P, C1P, and LPA, we did not observe any priming effect of LPC on the migration of A549 in response to an HGF/SF gradient. This is most likely the result of delayed conversion of LPC to LPA in an ATX-dependent manner.

These results demonstrate for the first time that chemokinetic factors such as BphsLs may prime or stimulate the migration of cells in reponse to chemoattractants, and this effect does not rely on simple synergistic effects.

### BphsLs enhance the prometastatic properties of LC cells by modulating the microenvironment

**Figure 5:**
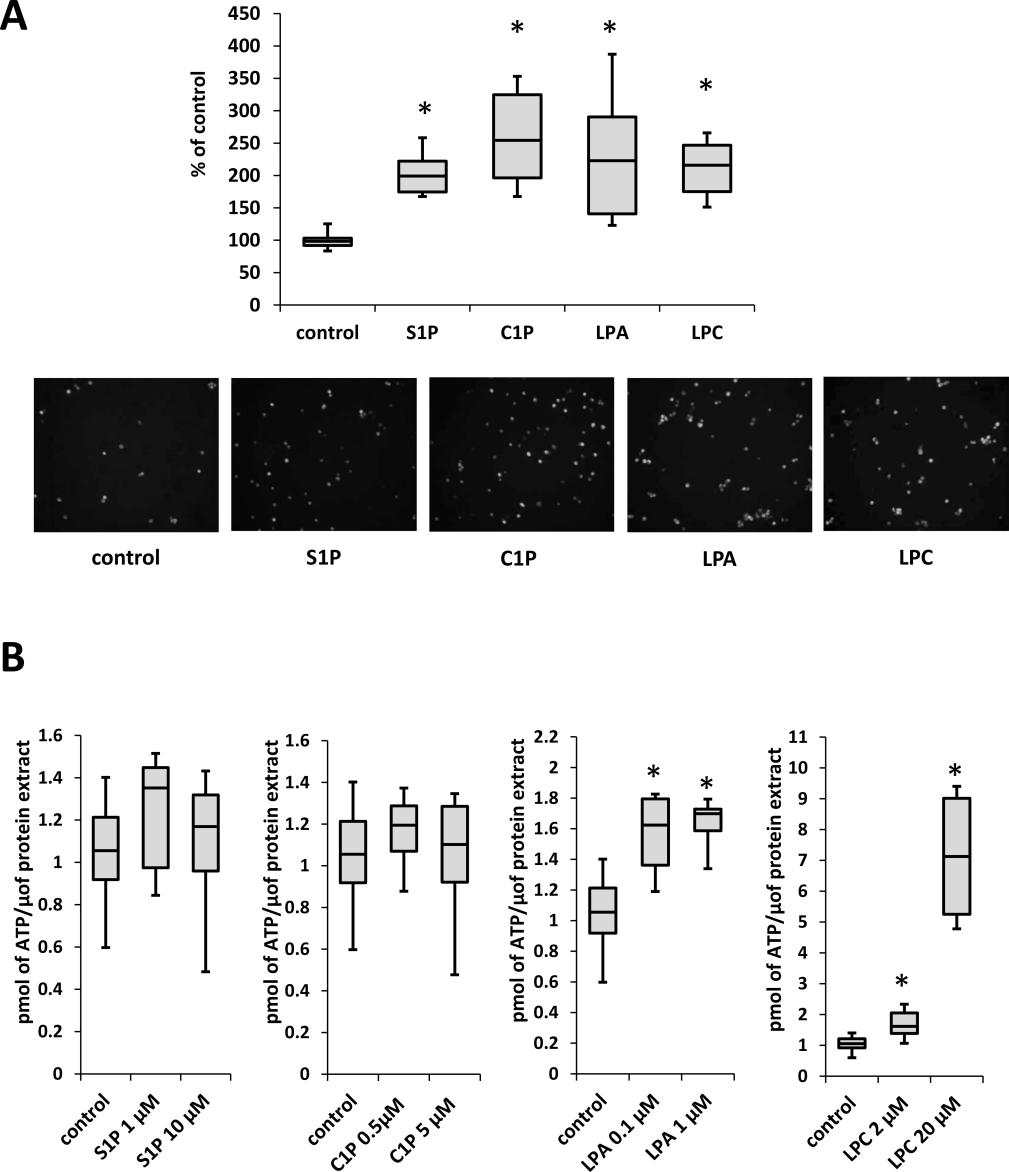
BphsLs regulate LC–stromal interactions (Panel **A**) The adhesion of A549 cells to human stromal cells. LC cells stained with calcein as well as stromal cells were stimulated with S1P (1 μM), C1P (0.5 μM), LPA (0.1 μM ), or LPC (20 μM) for 30 min. After a 20-minute incubation, non-adherent cells were removed, and adherent cells counted under a fluorescence microscope. Data from two separate experiments are pooled together, and means ± SD are shown, *p ≤ 0.05. Lower panel includes representative pictures of calcein-labeld A549 cells that adhered to stroma cells after stimulation (or not) with different BphsLs. (Panel **B**) The level of ATP in conditioned medium from stromal cells stimulated for 1 min with S1P, C1P, LPA, or LPC. Data from two separate experiments are pooled together, and means ± SD are shown, *p ≤ 0.05.

It is well known that the microenvironment can modulate the responsiveness of cancer cells, and the interaction of cancer cells with stroma is crucial for their survival. Therefore, we tested the effect of BphsLs on the interaction between LC cells and bone marrow stromal cells and found that all tested BphsLs, in particular C1P and LPA, increased adhesion of LC cells to stroma (Figure 5A).

Recently we identified ExNs as potent chemokinetic factors for LC cells [10]. We therefore became interested in whether BphsLs enhance ATP release from stromal cells. Indeed, we found that treatment of stromal cells with LPA and LPC, but not with S1P or C1P, increased the level of ATP in conditioned media harvested from these cells (Figure 5B).

### Bioactive lipids are upregulated after irradiation or chemotherapy as pro-migratory factors for LC cells

**Figure 6:**
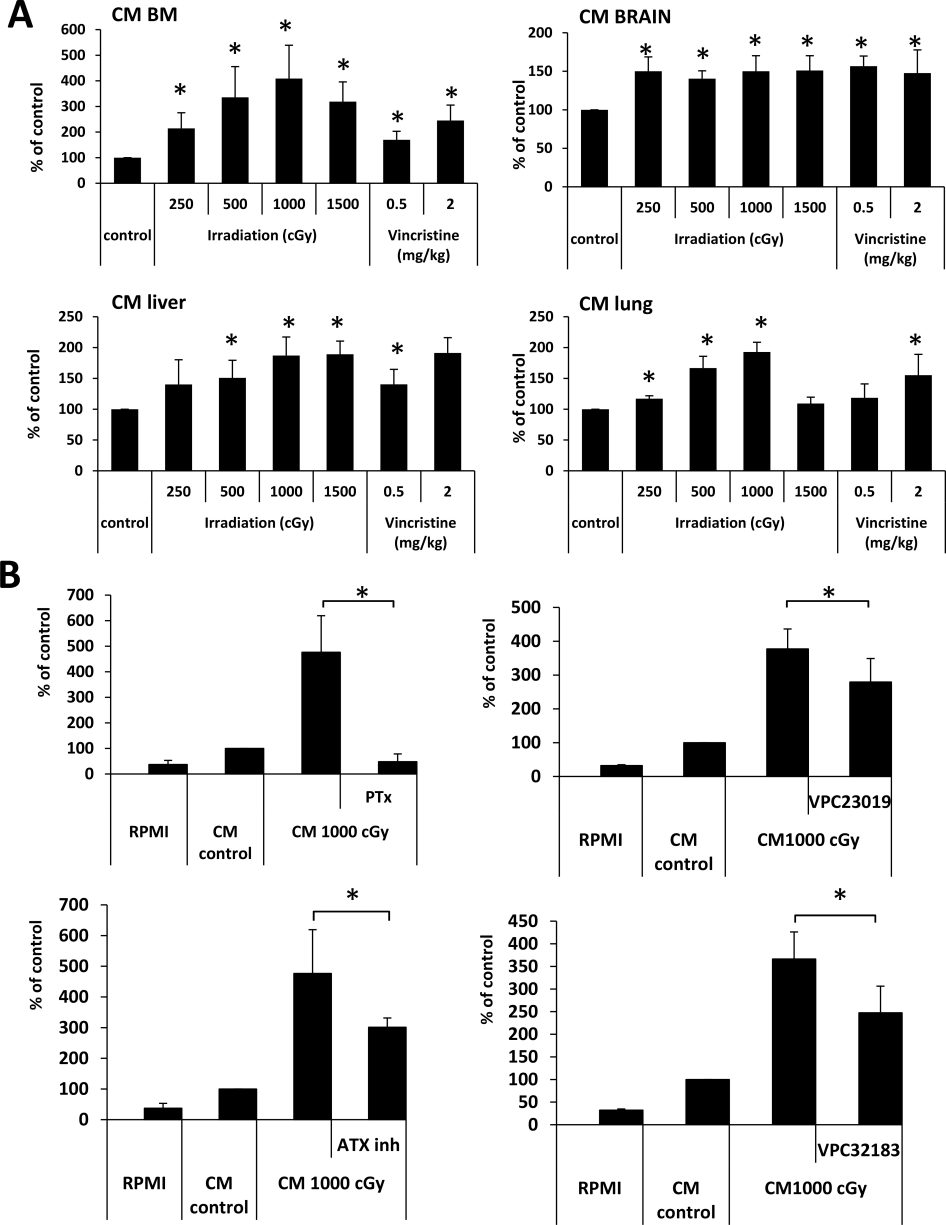
BphsL levels create a prometastatic microenvironment in irradiated organs (Panel **A**) Conditioned media (CM) from irradiated BM, liver, and lung cells enhance the migration of A549 cell lines across Transwell membranes. The results from two independent experiments are shown as means ± SD. *p ≤ 0.05 compared with the control (CM from cells from untreated animals). (Panel **B**) The effect of pertussis toxin (PTx, 1 μg/ml), VPC32183 (10 μM), VPC23019 (20 μM), and the autotaxin inhibitor S32826 (ATX inh, 10 μM) on the migration of A549 cells in response to CM from irradiated BM. The results from two independent experiments are shown as means ± SD. *p ≤ 0.05 compared with the control (CM from cells from untreated animals). Migration in response to RPMI is shown as reference.

As mentioned in the introduction, we have proposed that one of the unwanted side effects of radio- and chemo- therapy is induction of a prometastatic environment in different tissues [10, 13, 14]. In fact, we found high levels of BphsLs as well as ExNs in supernatants harvested from cell suspensions prepared from bone marrow, liver, lung, and brain [10, 13, 14]. To test whether supernatants from these organs induce motility of LC cells, we measured the responsiveness of A549 cells to these supernatants by employing the Transwell migration assay and found that these cells migrate in response to these supernatants (Figure 6A).

In our previous studies we found that S1P, C1P, LPA, and LPC [13, 14] as well as certain ExNs, including ATP [10], are present in conditioned media from organs exposed to radio- and chemo- therapy and play a role in increasing the motility of cancer cells. Here we became interested in the potential role of BphsLs in the induction of a pro-migratory response of LC cells to conditioned medium prepared from irradiated BM cells (Figure 6B).

Since BpshL receptors are G protein-coupled receptors, we first employed an inhibitor of G protein-coupled receptor signaling, pertussis-toxin (PTx), and found that pre-treatment of cells with PTx completely abolished their responsiveness to conditioned media harvested from irradiated BM cells (Figure 6B, left upper panel). We also employed a competitive inhibitor of S1P receptor types 1 and 3 (VPC23019), LPA receptor types 1 and 3 (VPC32183), or an ATX inhibitor (Figure 6B, upper right panel and lower panels respectively), and observed that LC motility was inhibited by ~25–30%.

Together, our results suggest that BphsLs significantly contribute to the increased responsiveness of LC cells to conditioned media prepared from irradiated or vincristine-exposed organs.

## DISCUSSION

The most important contributions of this paper are recognition of the pleiotropic effects of BphsLs in LC metastasis/progression, identification of C1P as a novel prometastatic factor for LC cells, and identifying the priming effect of chemokinetic factors in the response of cells to chemoattractants. All observed effects occurred at physiological concentrations of BphsLs normally present in biological fluids.

A major problem in cancer therapy is the recurrence of tumor growth after successful initial treatment and the tendency of cancerous cells to spread and metastasize to different vital organs [35, 36]. Recently, we proposed that the spread of tumors might be the result of the formation of a pro-metastatic environment as an unwanted side effect of radio- and chemo- therapy [10, 13, 14, 37]. Paradoxically, however, while treatment is necessary to kill or reduce the number of cancer cells, at the same time it results in collateral tissue or organ damage that leads to the release of several potent pro-migratory factors, such as chemokines, pro-migratory growth factors, ExNs, and BphsLs [10, 13, 14]. Therefore, cells that survive initial treatment become exposed to these strong pro-migratory signals, leave the site of the primary tumor, and spread to other tissues.

All BphsLs studied in this paper, including S1P, C1P, and LPA (together with its precursor LPC), are well-known factors that regulate cell growth, survival, and migration of different types of non-malignant and malignant cells [38–42]. In particular, S1P and LPA are the most-studied BphsLs in cancer biology. Sphingosine kinase 1 (SPHK1), an enzyme that regulates synthesis of S1P, was found to be upregulated in different types of lung cancer and described as being involved in migration and tumor progression [43]. In patients with astrocytoma, the same enzyme was also found to correlate with disease progression and reduced survival [44].

Similarly, the LPC derivative LPA [20], which is synthesized from LPC in an ATX-dependent manner, was also found to play a role in induction of the migratory response of several different types of cancer cells [45, 46]. In fact, all LC cell lines tested in our studies expressed ATX mRNA. Moreover, since LC cells also express LPC receptors, as in the case of rhabdomyosarcoma [14], T-cells [47], and macrophages [48], LC may also directly stimulate cell migration.

In this paper we show for the first time the involvement of C1P in the progression of LC. The biological role of C1P, in contrast to other BphsLs, has been somewhat ignored. The most recent studies have shown its effect on the migration of THP-1 [49], rhabdomyosarcoma [14], and pancreatic cancer cells [50]. Here we demonstrate for the first time the involvement of C1P in the metastasis of LC cells. In our hands, C1P enhanced the motility, adhesion, and interaction of LC cells with stroma. Importantly, LC cells responded to physiological concentrations of this BphsL. Unfortunately, studies on the role of C1P in cell migration are hampered because its receptors have not yet been cloned [19]. The known potent effects of C1P on tumor metastasis should prompt studies to identify C1P receptor/s and to develop inhibitors of this BphsL.

Several cooperating signaling pathway were found to play a role in regulation of migration and adhesion of both normal and malignant cells in response to BpshLs stimulation. Activation of e.g. MAPK1/2 AKT, p38, PI3K in response to S1P, LPA, LPC or C1P [13, 14, 19, 51–53] was found to be crucial for regulation cell migration since inhibition of these pathways resulted in decreased or even completely abolished cell migration. In our study LC cell lines responded to BpshLs by activation of MAPK1/2 and AKT signaling pathways, what suggests that these signaling pathways are important for migration of LC cells. It is well known that down-stream effects of BpshLs stimulation include activation of Rho family proteins such as RhoA and Rac1 which are regulators of actin cytoskeleton [50, 53, 54]. In addition, focal adhesion kinase (FAK), a highly conserved cytoplasmic tyrosine kinase involved in the engagement of integrin and assembly of focal adhesions sites is as reported effector of S1P stimulation [55]. LPA (and probably also LPC through its conversion to LPA by ATX) was found to activate mDia formins which on one hand induce actin polymerization, from the other hand stabilize microtubules [56, 57].

Overall, our results provide broader insight into several pleiotropic effects of BphsLs on the metastatic behavior of LC cells, including their influence on the biological effects of other potentially prometastatic factors. Recently, we identified ExNs as potent chemoattractants, with their levels being upregulated after radio- and chemo- therapy [10]. We therefore became interested in whether BphsLs modulate the secretion of ExNs from stromal fibroblasts and found that this is the case. Corroborating our observation, LPA has been reported to induce the secretion of ATP from microglia cells [58]. Interestingly, we observed an even more robust increase in ATP level after stimulation of cells with LPC. Due to the short time of stimulation (1 min), it seems that this effect of LPC is specific and not an ATX-dependent conversion of LPC to LPA.

As mentioned above, cell motility is a response to chemotactic and/or chemokinetic factors [34]. As demonstrated in our work, BphsLs induce, as in the case of rhabdomyosarcoma cells [13, 14], random migration of LC cells, a process known as chemokinesis. In this paper we investigated the potential relationship between both pro-migratory processes and report for the first time that chemokinetic factors may sensitize or prime the response of cancer cells to chemokinetic stimuli. This responsiveness is different from a synergic effect, and further studies are required to explain this effect at the molecular level. This effect may depend on membrane lipid raft formation [59], which assembles together receptors and signaling molecules for more efficient interactions, or on activation of complementary signaling pathways specific for each factor [60].

**Figure 7:**
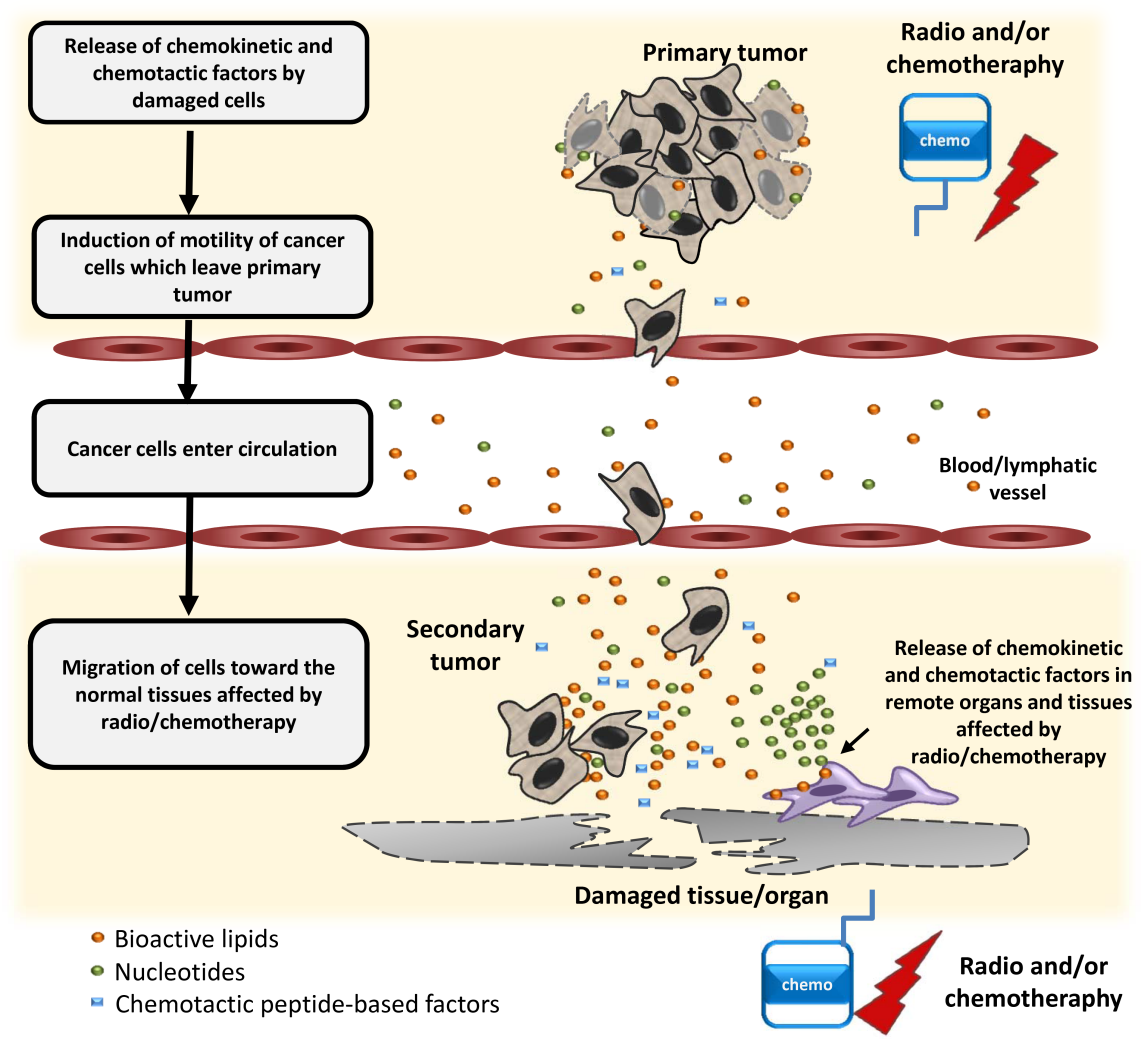
Schematic representation of the role of BphsLs, ExNs, and chemotactic peptides in the regulation of the metastasis of lung cells as a side effect of radio- and/or chemo-therapy

In summary, we provide novel evidence that radio- and/or chemo- therapy may induce a prometastatic environment for LC cells. As depicted in Figure 7, radio- and/or chemo-therapy-induced tissue or organ damage leads to the release in tumor and collateral healthy tissues of BphsLs and other pro-migratory factors that stimulate migration of LC cells that have survived initial treatment. This stimulation leads to egress of therapy-resistant cells from the primary tumor. It is worth stressing that, in comparison with chemokines and migration-enhancing growth factors, BphsLs and ExNs are expressed at a much higher and more biologically significant level in damaged tissues. Radio- and/or chemo- therapy also induces a prometastatic environment in several distant organs. In these distant locations, BphsLs steepen chemotactic gradients of chemokines and tumor-attracting growth factors. All these factors collectively enhance the seeding efficiency of cancer cells circulating in peripheral blood and lymph. In conclusion, our results show that BphsLs are important modulators of prometastatic environment. Therefore, their inhibitors could be considered as potential anti-metastatic drug candidates to be included as a part of post radio- and/or chemo- therapy treatment. This however, requires further studies.

## MATERIALS AND METHODS

### Cell lines

We used several human lung cancer cell lines (obtained from the American Type Culture Collection, Manassas, VA, USA), including both non-small cell lung cancer (NSCLC; A549, HTB177, HTB183, and CRL5803) and small cell lung cancer (SCLC; CRL2062 and CRL5853) cell lines. NSCLC cells were cultured in Roswell Park Memorial Institute (RPMI) medium 1640, containing 10% fetal bovine serum (FBS), 100 U/ml penicillin, and 10 μg/ml streptomycin. CRL2062 cells were maintained in Waymouth's MB 752/1 medium containing 10% FBS, 100 U/ml penicillin, and 10 μg/ml streptomycin. CRL5853 cells were cultured in DMEM/F12 medium supplemented with 5% FBS, 0.05 mg/ml insulin, 0.01 mg/ml transferrin, 30 nM sodium selenite (ITS, Lonza, Allendale, NJ, USA), 10 nM hydrocortisone (Sigma-Aldrich, St. Louis, MO, USA), 10 nM beta-estradiol (Sigma-Aldrich), 4 mM L-glutamine, 100 U/ml penicillin, and 10 μg/ml streptomycin. All cells were cultured in a humidified atmosphere of 5% CO_2_ at 37°C, and the media were changed every 48 hours.

### Murine bone marrow stroma cells

Bone marrow derived stromal cells were expaned *ex vivo* from murine bone marrow mononucler cells (BMMNC) as described [16]. Briefly, BMMNC were expanded in DMEM supplemented with 20% FBS and 50 U/ml penicillin/streptomycin for 7–10 days at 37°C in a 5% CO2 incubator. Cells between passage numbers 1–3 were used for experiments.

### Reverse transcriptase-polymerase chain reaction (RT-PCR)

mRNA was extracted and purified from cells using the RNeasy Mini kit (Qiagen Inc., Germantown, MD, USA) and treated with DNase I (Qiagen Inc.). The purified mRNA (2500 ng) was afterwards reverse-transcribed into cDNA using First Strand cDNA Synthesis (Thermo Scientific, Waltham, MA, USA). Amplification of synthesized cDNA fragments was carried out using AmpliTaq Gold polymerase (Applied Biosystems). The PCR conditions were: 1 cycle of 8 min at 95°C; 2 cycles of 2 min at 95°C, 1 min at 60°C, and 1 min at 72°C; 40 cycles of 30 s at 95°C, 1 min at 60°C, and 1 min at 72°C; and 1 cycle of 10 min at 72°C. The human sequence-specific primers are presented in Table 1.

**Table 1:** Sequences of primers employed for RT-PCR

RT-PCR primers		
Name	Forward primer sequence	Reverse primer sequence
S1PR_1_	GAACAGCATTAAACTGACCTCGG	CAAACATACTCCCTTCCCGC
S1PR_2_	CCGATTTCTCCTTTTCGAATGT	AAATAGTGCAACTGAGCAATGAGG
S1PR_3_	GCAGCACTTCAGAATGGGATC	GGCAGTGGATGATGTCAGCA
S1PR_4_	TTCTGACGCCAAATGGGC	GATCGAACTTCAATGTTGCCAG
S1PR_5_	CCCCACAATGTGAACAAACAGA	TCCCTCATCCTGAAATGCTTTT
LPAR1	GCAGCTCCACACACGGATG	TAGTCCTCTGGCGAACATAGCC
LPAR2	CTGTCGAGCCTGCTTGTCTTC	CAGCCTAAACCATCCAGGAGC
LPAR3	TGTCTCCGCATACAAGTGGG	GGGTTCACGACGGAGTTGAG
LPAR4	TACAACTTCAACCGCCACTGG	ACATTAGTGGTGGAAAACAAAGAGG
LPAR5	GATGAAGCTGTGACCAAACGC	CATGGTCCCAAAACAAGCAGA
G2A	GAGCGTCTGTCAGCGGAGTC	CGTGTGCCAGGATTTCCCT
GPR4	CCACATTGCCTGAACTTTCCA	TGGAGTCAGTGTGTCAACGAGG
SK1	TGAGCAGGTCACCAATGAAG	GGCTGAGCACAGAGAAGAGG
SK2*	ACAACGAGGAGAGCTGCAAT	AGAAGTCCAGGCTGGTGAGA
SPP1	ATTCATCATCATCGGCTTC	CAATTCCAGCACCACTTCCT
SPP2	ATGCATACGGTCCTGGATGT	TATGACACACACGGGGAAGA
S1p lyase	ATACTGATGGCCTGCAAAGC	TCCCAAAGTAACTGGCTGCT
CERK	GAGAAGCTGACGTCCAGACC	CCTTGGCCTGATTAGCATGT
LPP1	AGGGAGCTCTGGTTGCAATA	TCCCAGTTGTTGGTGTTTCA
LPP2	TACACCCGCGTGTCTGATTA	TCCTTCAGACAGTGCTGTGG
LPP3	CCTCATCATCGAGACAAGCA	CACAGAGCACAGCGTCATTT
autotaxin	AAGGCAAAGAGAACACGCTG	ATCTGACACGACTGGAACGAG
β-actin	GGATGCAGAAGGAGATCACTG	CGATCCACACGGAGTACTTG

*annealing temperature 58°C (instead of 60°C as for all others primers sets)

### Phosphorylation of intracellular pathway proteins

The A549 and CRL5853 cell lines were kept overnight and for 6 h, respectively, in medium containing 0.5% BSA to render the cells quiescent. The cells were then stimulated with bioactive lipids or vehicle only (control cells) at 37°C for 5 min (for S1P, C1P and LPA) or 2 h (for LPC), then lysed for 20 min on ice in RIPA lysis buffer containing protease and phosphatase inhibitors (Santa Cruz Biotech, Santa Cruz, CA, USA). The extracted proteins were separated on a 12% SDS-PAGE gel and transferred to a PVDF membrane. Phosphorylation of the serine/threonine kinase AKT (phospho-AKT473) and p44/42 mitogen-activated kinase (phospho-p44/42 MAPK) was detected by rabbit antibodies (cat # 9101 and 9271 respectively, both from Cell Signaling, Danvers, MA, USA), with HRP-conjugated goat anti-rabbit and anti-mouse secondary antibodies (cat # 7074 Cell Signaling). Equal loading in the lanes was evaluated by stripping the blots and reprobing with anti-p42/44 MAPK monoclonal antibody (clone no. 9102, cat # 4696, Cell Signaling) with HRP-conjugated goat anti-mouse secondary antibodies (cat # 7076, Cell Signaling). The membranes were developed with enhanced chemiluminescence (ECL) reagent (Amersham Life Sciences, Arlington Heights, IL, USA), dried, and subsequently exposed to film (Hyperfilm, Amersham Life Sciences). Results from three independent experiments ware quantified using ImigeJ Software. Values obtained for bands representing phosphorylated proteins were relativized to bands representing total protein level.

### Chemotaxis and chemokinesis assays

Chemotaxis assays were performed in a modified Boyden's chamber with 8-μm polycarbonate membrane inserts (Costar Transwell; Corning Costar, Lowell, MA, USA). In brief, cells detached with 0.25% trypsin were made quiescent by incubation for 1–3 h in appropriate medium (RPMI, DMEM-F12, or Waymouth's MB 752/1), supplemented with 0.5% (for NSCLC cells) or 0.2% (for SCLC cells) bovine serum albumin (BSA). The cells were then seeded into the upper chamber of an insert (pretreated with 1% gelatin) at a density of 4 × 10^4^ (NSCLC) or 10 × 10^4^ (SCLC) cells in 110 μl. The lower chamber was filled with pre-warmed medium containing test reagents. After 24 hours, the inserts were removed from the Transwell supports. The cells that had not migrated were scraped off with cotton wool from the upper membrane, and the cells that had transmigrated to the lower side of the membrane were fixed and stained with HEMA 3 (protocol, Fisher Scientific, Pittsburgh, PA, USA) and counted on the lower side of the membrane using an inverted microscope. In some experiments cells were pretreated with pertussis toxin (PTx) for 1 h or with the inhibitors VPC32183 or VPC23019 for 30 min before the chemotaxis experiment.

In chemokinetic experiments, BsphLs were added in the same concentration to both, upper and lower chambers, therefore no gradient was created but the LC cells were exposed to BsphL. In priming experiments, cells were pretreated with studied BsphLs and loaded to upper chamber. Lower chambers contained medium with or without HGF.

### Cell proliferation

Cells were seeded in culture flasks at an initial density of 1.25 × 10^4^ cells/cm^2^ (NSCLC cells) or 6 × 10^4^ cells/cm^2^ (SCLC cells). After 24 h, the medium was changed to new medium supplemented with 0.5% BSA, and the cells were cultured in the presence or absence of BsphLs. Full medium (with FBS) was treated as a positive control. The cell number was calculated at 24, 48, and 72 h after the change of medium. At the indicated time points, cells were harvested from the culture plates by trypsinization and counted using Trypan Blue and a Neubauer chamber.

To evaluate if BpshLs may protect LC to vincristine treatment, cells were plated in culture flasks at an initial density of 2.25 × 10^4^ cells/cm^2^. After 24 h, the culture medium was changed to new RPMI supplemented with 0.5% BSA, and cells were cultured in the presence or absence of S1P (1 μM), C1P (0.5 μM), LPA (0.1 μM) or LPC (20 μM) with or without vincristine (0.5 or 5 μM). In control experiments cells were cultured in RPMI supplemented with 0.5% BSA. The cell number was calculated at 24, 48, and 72 h after the change of medium. At the indicated time points, cells were harvested from the culture plates by trypsinization and stained using Trypan Blue and counted in Neubauer chamber.

### Adhesion assay to fibronectin

Cells were made quiescent for 3 hours with appropriate medium containing BSA (0.5% or 0.2%) and incubated with nucleotides for 10 min. Subsequently, cell suspensions (5 × 10^3^/100 μL) were added directly to 96-well plates coated with fibronectin and incubated for 5 min at 37°C. The wells were previously coated with fibronectin (10 μg/ml) overnight at 4°C and blocked with 0.5% BSA for 1 hour before the experiment. Following incubation, the plates were vigorously washed three times to remove non-adherent cells, and the adherent cells were counted using an inverted microscope.

### Adhesion to stromal cells

Stromal cells have been plated in 96 well plates and used in experiments when reach 95–100% confluence. A549 cells were labeled before assay with the fluorescent dye calcein AM. Next, both stromal and A549 cells were made quiescent by incubation for 3 h at 37°C in RPMI 1640 medium supplemented with 0.5% BSA, followed by stimulation with BsphLs for 30 min at 37°C. A549 cells were then added to plates with stromal cells and incubated for 20 min at 37°C. After this the non-adherent cells have been discarded, and cells that adhered to the stromal cells were counted under a fluorescence microscope. During data collection we performed additional analysis in a bright field to confirm that fluorescent-stained cells are attached to stroma and not to the plastic.

### Quantitation of ATP

The ATP levels secreted by stroma into the medium were measured using the ATP Colorimetric/Fluorometric Assay kit and Deproteinizing Sample Preparation kit (BioVision), according to the manufacturer's protocol. Fluorescence analysis was performed with Ex/Em set at 535/585 nm. Briefly, cells were seeded into 48-well microplates (2 × 10^5^ cells per well) and allowed to attach. Cells were kept quiescent for 24 h by incubation in DMEM supplemented with 0.5% BSA, followed by a 1-min stimulation with bioactive lipids in DMEM alone. Medium was collected, the cells were lysed in RIPA buffer, and the protein concentration was measured using a BCA Protein Assay (Thermo Scientific). All ATP calculations were performed per 1 μg of protein in the cell extract.

### Preparation of conditioned media

Pathogen-free C57BL/6 mice were purchased from the National Cancer Institute (Frederick, MD, USA), allowed to adapt for at least 2 weeks, and used for experiments at age 7–8 weeks. Animal studies were approved by the Animal Care and Use Committee of the University of Louisville (Louisville, KY, USA). Mice were irradiated with 250, 500, 1000, or 1500 cGy. Twenty-four hours later, bone marrow and plasma were isolated. Conditioned media (CM) obtained by 15 min incubation of BM cells or liver, brain and lung tissues (mechanically homogenized using scissors and/or syringe) in RPMI at 37°C. After centrifuging, the supernatant was used for further experiments. In studies with the chemotherapeutic agent vincristine, mice were injected intraperitoneally with 0.9% NaCl with (0.5 mg/kg or 2 mg/kg) or without vincristine. Twenty-four hours later, the organs were isolated, and CM was prepared as described above.

### Statistical analysis

Statistical analysis of the data was done using the *t*-test (for data having a normal distribution) or the Whitney–Mann test (for data not having a normal distribution), with *p* < 0.05 considered significant.

## SUPPLEMENTARY MATERIALS FIGURES



## Abbreviations

ATX: autotaxin
BphsLs: bioactive phospholipids
C1P: ceramide-1-phosphate
CCL19: chemokine (C-C motif) ligand 19
ExNs: extracellular nucleotides
HGF/SF: hepatocyte growth factor/scatter factor
LC: lung cancer
LPA: lysophosphatidic acid
LPC: lysophosphatidylcholine
MCP1: monocyte chemoattractant protein 1
SDF-1: α-chemokine stromal-derived factor 1
PTx: pertussis toxin
