# Supplementary material for: Novel pleiotropic effects of bioactive phospholipids in human lung cancer metastasis
Source: Oncotarget. 2017 Apr 27;8(35):58247–63. doi: 10.18632/oncotarget.17461 (PMC5601648; doi:10.18632/oncotarget.17461)
Supplement: Supplementary file 1 [file oncotarget-08-58247-s001.pdf]

Novel pleiotropic effects of bioactive phospholipids in human lung cancer metastasis

Supplementary Materials

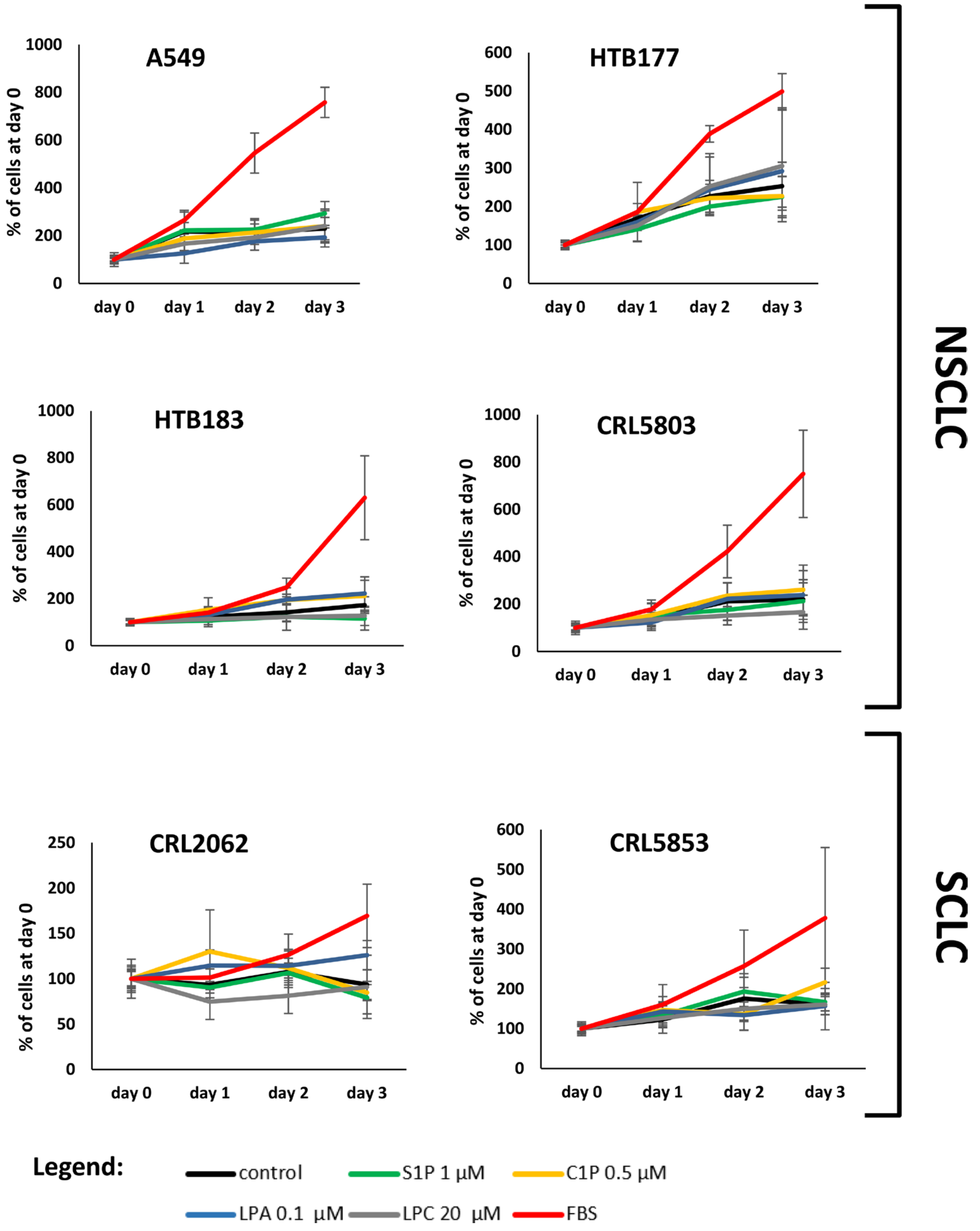

Supplementary Figure 1: BphsLs do not induce migration of LC cell lines in serum free conditions. Analysis of proliferation of LC cells in the presence or absence of S1P, CIP, LPA and LPC. FBS served as a positive control. Experiment was performed twice in at least duplicates.

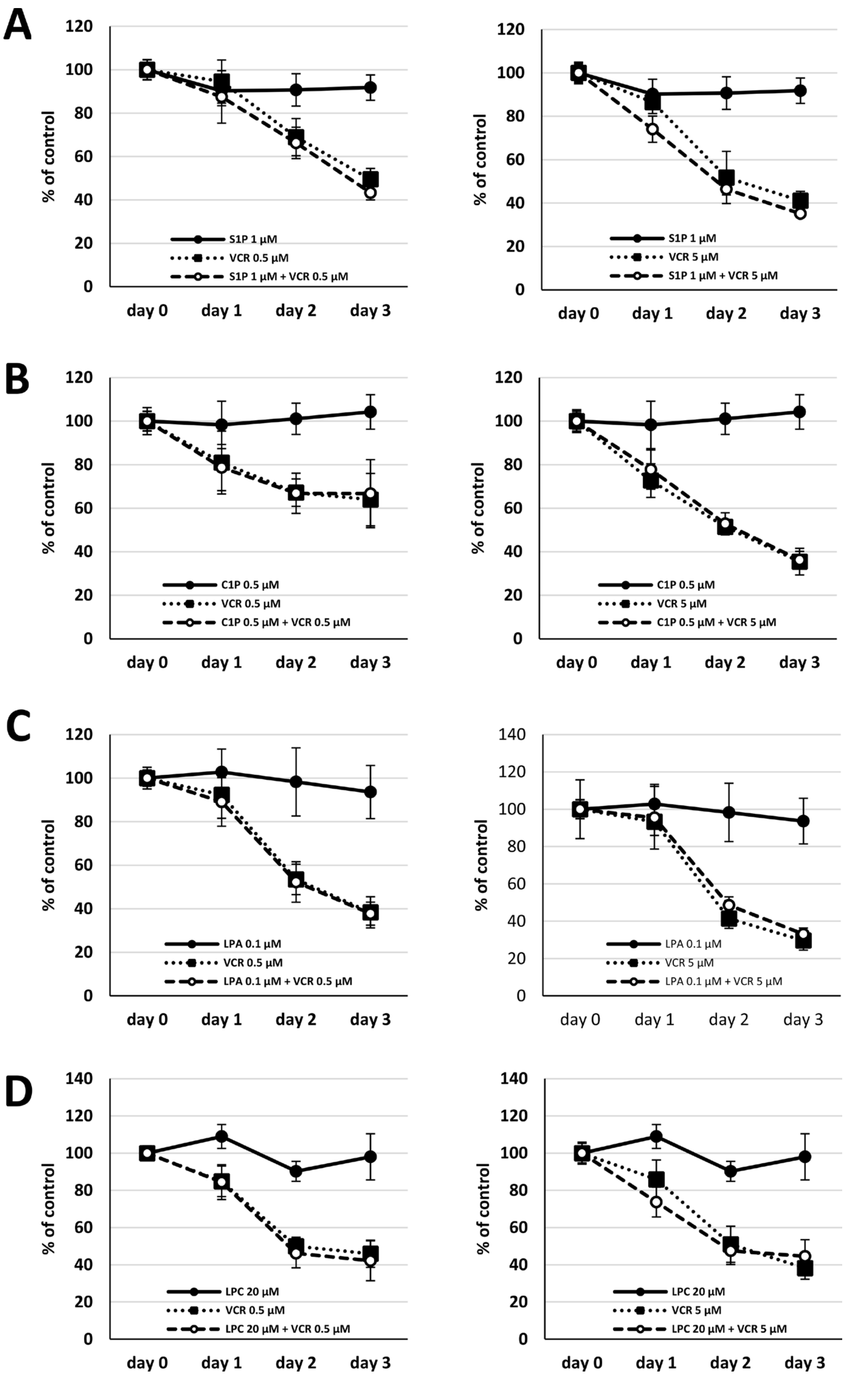

Supplementary Figure 2: BphsLs do not enhance the survival of LC cells cultured under serum-free conditions in the presence of vincristine. Effect of S1P (Panel **A**), C1P (Panel **B**), LPA (Panel **C**) and LPC (Panel **D**) on survival of A549 cells treated with low (left panels) or high (right panels) dose of vincristine. Experiment was performed three times in triplicates.
